# How do students of different self-efficacy regulate learning in collaborative design activities? An epistemic network analysis approach

**DOI:** 10.3389/fpsyg.2024.1398729

**Published:** 2024-07-26

**Authors:** Peng Chen, Dong Yang, Jari Lavonen, Ahmed Hosny Saleh Metwally, Xin Tang

**Affiliations:** ^1^College of Education, Capital Normal University, Beijing, China; ^2^College of Education for the Future, Beijing Normal University, Zhuhai, China; ^3^Department of Education, University of Helsinki, Helsinki, Finland; ^4^Educational Technology Department, Faculty of Education, Helwan University, Cairo, Egypt; ^5^School of Education, Shanghai Jiao Tong University, Shanghai, China

**Keywords:** self-efficacy, self-regulated learning, collaborative design activities, epistemic network analysis, characteristics and developmental trajectories

## Abstract

**Introduction:**

Students' self-regulation skills and self-efficacy are linked to performance and are considered essential for lifelong learning. Understanding these skills and their development is crucial for educational success and long-term personal growth.

**Methods:**

In this study, 60 students attending a university-level collaborative design course were recruited as participants. They were initially classified into three groups [high, mixed, and low self-efficacy (SE)] based on the initial test results. Students' written reflections were then analyzed using epistemic network analysis (ENA), aiming to explore the characteristics and developmental trajectories of self-regulated learning (SRL).

**Results:**

Comparing with the other two groups, the high self-efficacy (HSE) group demonstrated: (1) more behavioral characteristics of SRL in the performance and self-reflection stages, (2) an earlier development of interest 91 in the task and recognition of its value during collaborative design activities, 92 followed by the utilization of more cognitive and metacognitive strategies; and (3) an “anticipation-behavior-reflection” loop in the self-regulation process.

**Discussion:**

These findings highlight the importance of fostering high self-efficacy among students to enhance their self-regulated learning capabilities and overall academic performance. Strategies for improving learners' SRL and future research directions were provided accordingly.

## 1 Introduction

Collaborative and active design thinking projects are effective ways to foster the development of participants' twenty-first-century skills, such as problem-solving, creativity, and collaboration (Luka, 2014). Collaborative learning is an educational strategy in which learners engage in group activities to address challenges, accomplish tasks, or create products (Laal and Laal, 2012). Typical elements of collaborative learning include common goals, interpersonal and group skills, and group processing, among others (Laal and Laal, 2012; Nokes-Malach et al., 2015). In collaborative learning, learners must engage more in high-level cognitive and metacognitive strategies and be able to strategically govern learning, as tasks require them to confront difficulties in real-world settings, cooperate to carry out a sequence of actions, and ultimately build inventive solutions. Therefore, self-regulated learning (SRL) is essential for students engaging in collaborative activities. SRL is a dynamic, positive process where learners define learning objectives and make an effort to regulate, monitor, and manage their behavior, cognition, and motivation to achieve certain learning objectives (Zimmerman and Schunk, 2008; Zimmerman, 2013). Growing evidence suggests that SRL skills play a significant role in managing the learning process and challenges and influence learning outcomes (e.g., Efklides, 2011; Teng et al., 2022).

Besides SRL, self-efficacy (SE) has been intensively investigated by academia based on evidence suggesting that students' SE promotes and helps maintain SRL skills in collaborative learning settings (Usher and Pajares, 2008b; Wilson and Narayan, 2016; Zhao and Cao, 2023). SE for learning is defined as a student's confidence in their capacity to study or carry out assignments successfully (Usher and Pajares, 2008a). Bandura (1977) states that efficacy beliefs help learners establish SR mechanisms that direct their goal-oriented behaviors across time and in the face of changing circumstances. Effective planning, goal formulation, and cautious task selection are necessary for the collaborative competency (Aguado et al., 2014); variable degrees of collective efficacy might have a knock-on impact, eventually altering the link between collective efficacy and satisfaction. Typically, learners with high levels of SE use more cognitive and metacognitive strategies, study harder, and display greater persistence amid academic challenges (Vandevelde et al., 2015; De Backer et al., 2022; Chen et al., 2024).

The current study of SRL strategy use and SE in contexts of collaborative problem-solving or collaborative design has been relatively infrequent. A few studies have directly examined the relationship and found positive associations between learners' SE and the deployment of SRL strategies (e.g., De Backer et al., 2022; Fraile et al., 2023; Wang, 2023). In a recent study, Fraile et al. (2023) investigated how performance in a collaborative learning setting, self-regulation, and SE were affected by scripts and rubrics. However, their main goal was to contrast how using scripts and rubrics in group econometrics activities affected students' views, academic performance, self-regulation, and SE. Moreover, students of different SRL and SE levels in collaborative learning have been primarily explored in the computer-supported collaborative learning (CSCL) context (e.g., Cleary et al., 2017; De Backer et al., 2022). Typical studies of this scope tend to examine factors such as SRL and SE in collaborative learning within SRL-SE-performance trajectories across various disciplines, such as mathematics (Cleary et al., 2017), computer science (Paraskeva, 2007), medical education (Moghadari-Koosha et al., 2020), or English writing (Bai et al., 2021; Shen and Bai, 2022). While these studies mainly uncover positive effects on SRL, the results regarding the influence of SE and performance are mixed. So, further research is needed, particularly regarding the characteristics and developmental trajectories of SRL among students of different SE levels.

In educational psychology and learning analytics, especially in collaborative learning environments, extensive research has established connections between SRL strategies and achievement (Harris and Graham, 2009; Schunk and Zimmerman, 2013). However, some SRL researchers have raised concerns regarding the suitability of using self-report questionnaires as the main method of evaluating the application of strategies and behaviors associated with SRL (Winne et al., 2002). Researchers are starting to take into consideration alternative SRL assessment tools, such as direct observations or traces, think-aloud protocols, and teacher rating scales, due to the limitations of traditional coding-and-counting-based strategies, such as ignoring the temporal nature of verbal data and providing limited, and potentially misleading information about collaborative learning activities. Recent scholars have started to use epistemic network analysis (ENA) to investigate temporal proximity, and more especially temporal co-occurrences of codes, as a more suitable means of characterizing socio-cognitive learning processes in collaborative learning environments (Wu et al., 2023; An and Zhang, 2024). In addition, a social epistemic network signature has been suggested for examining the cognitive and social aspects of collaborative learning (Gašević et al., 2019). Therefore, in this study, we utilized the affordances of the ENA approach to explore students' SRL and SE during collaborative design activities. Specifically, the study categorized 60 undergraduates participating in a design thinking course into high-efficacy, mixed-efficacy, and low-efficacy groups based on cluster analysis. Then, we used the learners' reflective texts on their participation in collaborative design activities as a data source. Finally, we used ENA to explore the characteristics and developmental trajectories across the three different efficacy groups in terms of their SRL. Specifically, we ask: 1. What are the frequency distribution differences of SRL strategies among different SE groups? 2. What are the differences in SRL patterns among different SE groups? and 3. How does the SRL trajectory of different SE groups change in collaborative learning? It is worth mentioning that, despite design thinking being the context of collaborative learning, we do not focus on the design thinking learning process in this study. Instead, we focus on the SRL process and how it varies across different SE student groups.

## 2 Literature review

### 2.1 The multi-faceted SRL

SRL is an essential skill for lifelong learning (EU Council, 2002). Studies have found strong evidence that children's self-regulation in early school years is positively associated with later academic performance and negatively associated with depressive symptoms, substance abuse, and even physical illness in later school years and adulthood (Robson et al., 2020). One sharp feature of SRL is the development of models across these years. For example, Boekaerts is one of the earliest researchers in the SRL who approached SRL through a dual processing model (top-down/bottom-up) (Boekaerts and Niemivirta, 2000). Winne and Hadwin (2008) developed the SRL model, which emphasized goal orientation as a part of the use of metacognition in learning. However, one of the most popular SRL models is Zimmerman's (2000) cyclical phase model, where SRL is organized into three phases: forethought, performance and self-reflection. During the forethought phase, students analyze the situation, set objectives, and plan on how to accomplish them. Several motivating beliefs (i.e., SE) promote the process and influence the utilization of learning strategies during the performance phase. Finally, they assess their performance on the task and attribute responsibility for their success or failure. These conclusions elicit self-responses that might either positively or negatively influence how students approach the assignment in future studies. The cyclical phase model has been evaluated in several researches (e.g., Cleary et al., 2006; DiBenedetto and Bembenutty, 2013), which provides strong evidence for the validity of this model.

By adding monitoring stages to Zimmerman's model of SRL, Pintrich (2000) proposes a four-stage model, each stage containing four distinct regulatory domains: cognition, motivation/emotion, behavior, and environment. Meanwhile, learning strategies such as refinement and critical thinking are added to describe behaviors. The four-stage model highlights the role of motivation in SRL, and the Motivated Strategies for Learning Questionnaire (MSLQ) developed by Pintrich to measure students' SRL and SE. It is still widely used by researchers. This combination of phases and domains provides a thorough picture incorporating several SRL activities, such as activating prior knowledge, effectiveness assessments, and self-observations of behavior (Panadero, 2017).

The conceptual models of SRL have changed over the past few decades, shifting their emphasis to different facets of learning (Dignath et al., 2008). However, there are similarities. For instance, researchers agree that SRL comprises different stages and sub-processes, and most models are concretized around the three identifiable stages of planning, action, and evaluation/reflection. Meanwhile, those models present different phases and subprocesses (Panadero, 2017). Boekaerts' model focuses on the motivational aspects of regulated learning. At the same time, Winne and Hadwin (2008) constructed their model using information processing theory as a foundation, focusing on the efficient use of cognitive techniques to improve learning. The model developed by Hadwin et al. (2018) considers both individual regulation and the potential for shared regulation of learning among group members. Because Pintrich and Zimmerman's models emphasize motivation and metacognition as essential components of regulated learning, they might be categorized as broad models of regulated learning. In this study, the SRL model proposed by Zimmerman and Pintrich is used to analyze learners' SRL characteristics and development trajectories. We use those models since they fit the collaborative design learning context, where critical thinking, constant monitoring, and reflections are crucial for task success. For example, learning strategies such as refinement and critical thinking are included to describe behaviors in Pintrich's four-stage model.

### 2.2 The interaction between SRL and SE

SRL is often conceptualized as a multifaceted process including intentional attempts to regulate behavior, cognition, and metacognition in order to maximize learning within a specific situation (Pintrich, 2000; Zimmerman, 2000). Students' motivational beliefs, such as interest and SE, and their employment of self-regulation techniques to improve their performance are two essential elements of the majority of SRL models. From a social-cognitive standpoint, motivation beliefs are seen to be the main drivers of students' attempts to accomplish their objectives (Pintrich, 2000). According to Boekaerts and Cascallar (2006), students' SE beliefs affect their goals and the effort they put into their performance. Empirical research has supported the positive connection between SE beliefs and the application of SRL strategies (Magogwe and Oliver, 2007; Diseth, 2011; Kim et al., 2015).

Significant relationships between SE beliefs and SRL strategies were found across both K-12 and higher education settings (Magogwe and Oliver, 2007), for example, in the Norwegian sample (Diseth, 2011) and among South Korean college students (Yun et al., 2018). Moreover, most extant research on relations between SRL and SE has focused on how regulation profiles differ in SE for learning (e.g., Chen et al., 2022; De Backer et al., 2022), not vice versa. For instance, De Backer et al. (2022) found three distinguished profiles of regulators; second language learners' SRL strategies differed significantly across SE profiles in the computer-supported collaborative language learning environment. After conducting latent profile analysis (LPA) on English as a foreign language (EFL) students, Chen et al. (2022) found three SRL profiles that varied quantitatively in terms of SE levels. While most of the abovementioned studies concerned how students' SRL profiles differed across the SE levels, limited research was conducted on how students of various SE levels differ in SRL development (Kim et al., 2015). Therefore, this study aims to explore how students of different SE levels differ in terms of patterns and development of SRL strategies in an authentic, collaborative design activity.

### 2.3 Measuring issues of students' SRL in a collaborative learning environment

Typical quantitative analysis approaches such as structural equation modeling (SEM) and LPA tend to be performed on larger sample sizes. Although various approaches were used to measure students' SRL in collaborative learning, scholars are also worried about the potential risks of depending too much on quantitative analysis and its drawbacks in working with qualitative data (e.g., Wang et al., 2023). In quantitative discourse analysis, utterances are often coded, and variations in code frequency between circumstances are compared. However, they ignored the temporal character of speech data and provided limited and perhaps inaccurate information regarding collaborative learning processes (Csanadi et al., 2018). It is challenging to comprehend, support, and encourage the process of regulated learning in collaborative activities using standard instruments and methodologies (e.g., Järvelä et al., 2019; Zhang et al., 2021). Moreover, for some SRL studies with small sample sizes and manually coded interactions, new approaches are required to track the evolving development of regulatory mechanisms in various learning environments, providing insights for effective learning design considering the cyclical and dynamic of regulation (Zhang et al., 2021). Therefore, it is imperative to adopt new methodologies that can track the evolving development and formation of regulatory mechanisms within and between phases of collaborative learning (Malmberg et al., 2015).

Luckily, analysis approaches to understand the cyclical and dynamic nature of regulation have improved thanks to advancements in research and knowledge of self-and social regulation of learning, such as sequential pattern mining on log data and text mining on chat messages (Järvelä et al., 2019; Noroozi et al., 2019). Over the past decade, network models depicting phenomena that are both temporally and interdependent connected have been constructed using epistemic network analysis (ENA). This anthropological method quantitatively models discourse in various activities (Csanadi et al., 2018; Elmoazen et al., 2022). Due to the flexibility of the ENA method and the availability of free and user-friendly tools, it has been employed to model and explore complicated socioemotional phenomena in fields such as the social and cognitive facets of collaborative learning and the complex process of collaborative problem-solving (Zhang et al., 2021; Elmoazen et al., 2022).

In addition, content analysis is frequently employed to examine the knowledge construction (Gunawardena et al., 1997), cognitive presence (Garrison et al., 2001), and SRL in collaborative learning by analyzing discussion transcripts generated in the learning process. Considering its flexibility and functionality in dealing with quantitative ethnographic data and the dynamic nature of self-regulation, we in this study opted for the ENA approach and content analysis to understand the patterns and development trajectories of SRL among undergraduates of different SE levels during various stages of collaborative design activities. The next section describes the context and research design we applied, along with the analysis plan.

## 3 Research design

### 3.1 Research context and collaborative design tasks

#### 3.1.1 Research context

This study was conducted as part of a university-selective course titled *Design Thinking and Innovation Design* during Autumn 2022–2023. There was no grade limit for enrollment. The course lasted for 12 weeks and aimed to assist students in how to collaboratively and creatively solve problems through the design thinking process. Students completed tasks in groups and were required to submit a design scheme and prototype to adhere to the theme “future school design.”

#### 3.1.2 Activity design

In this study, students completed a design project following the design thinking process and methods (depicted later in this section). Design thinking is a curious, creative, collaborative, human-centered approach to problem-solving that appreciates many viewpoints on an issue (Dunne and Martin, 2006; Brown, 2008; Melles and Misic, 2011).

Over the past decades, dozens of design thinking models have been developed. Simon (1969) introduced the first design thinking model, consisting of a sequential process with three stages: analysis, synthesis and evaluation. Brown (2008), the CEO of IDEO, suggested that design thinking involves three stages that work in a circular mode: inspiration, creativity, and realization. In addition, the EDIPT model of design thinking developed by Stanford University School of Design entails five stages: empathy, definition, idea, prototype, and test (Hassi and Laakso, 2011), which has been most commonly used in a variety of disciplines.

We synthesized the three-stage design thinking model of the EDPIT model. The task was divided into three stages: inspiration-ideation-realization, including six sub-tasks (empathy, problem definition, ideation, prototype, test, and iteration). The first stage was inspiration, which included two sub-tasks; one was to empathize and understand users, and the other was to define the problem to be solved in the project. The second stage was ideation, where students needed to explore the solution and design scheme for the initial question and then make prototypes with various materials and approaches. Finally, at the realization stage, the solutions/prototypes must go through rounds of tests and iteration for perfection.

### 3.2 Participants

There were sixty participants in the study, consisting of 42 females and 18 males. The participants ranged from 18 to 21 years old, with an average age of 19.03. The participants consisted mainly of first- and second-year college students, with a smaller number of juniors and seniors. They came from different majors, such as math, physics, information engineering, foreign language, management, literature, and arts. A detailed description of the participants is provided in Table 1.

**Table 1:** Participants' profile.

**Gender**		**Age**		**Years of university**		**Major**	
Females	42 (70%)	18	18 (30%)	1	22 (36.67%)	Music	24 (40%)
Males	18 (30%)	19	26 (43.33%)	2	34 (56.67%)	Arts	10 (16.67%)
		20	12 (20%)	3	2 (3.33%)	Physics	8 (13.33%)
		21	4 (7.67%)	4	2 (3.33%)	Math	6 (10.00%)
						Management	4 (6.67%)
						Literature	4 (6.67%)
						Information	2 (3.33%)
						Foreign language	2 (3.33%)

### 3.3 Procedures

**Figure 1:**
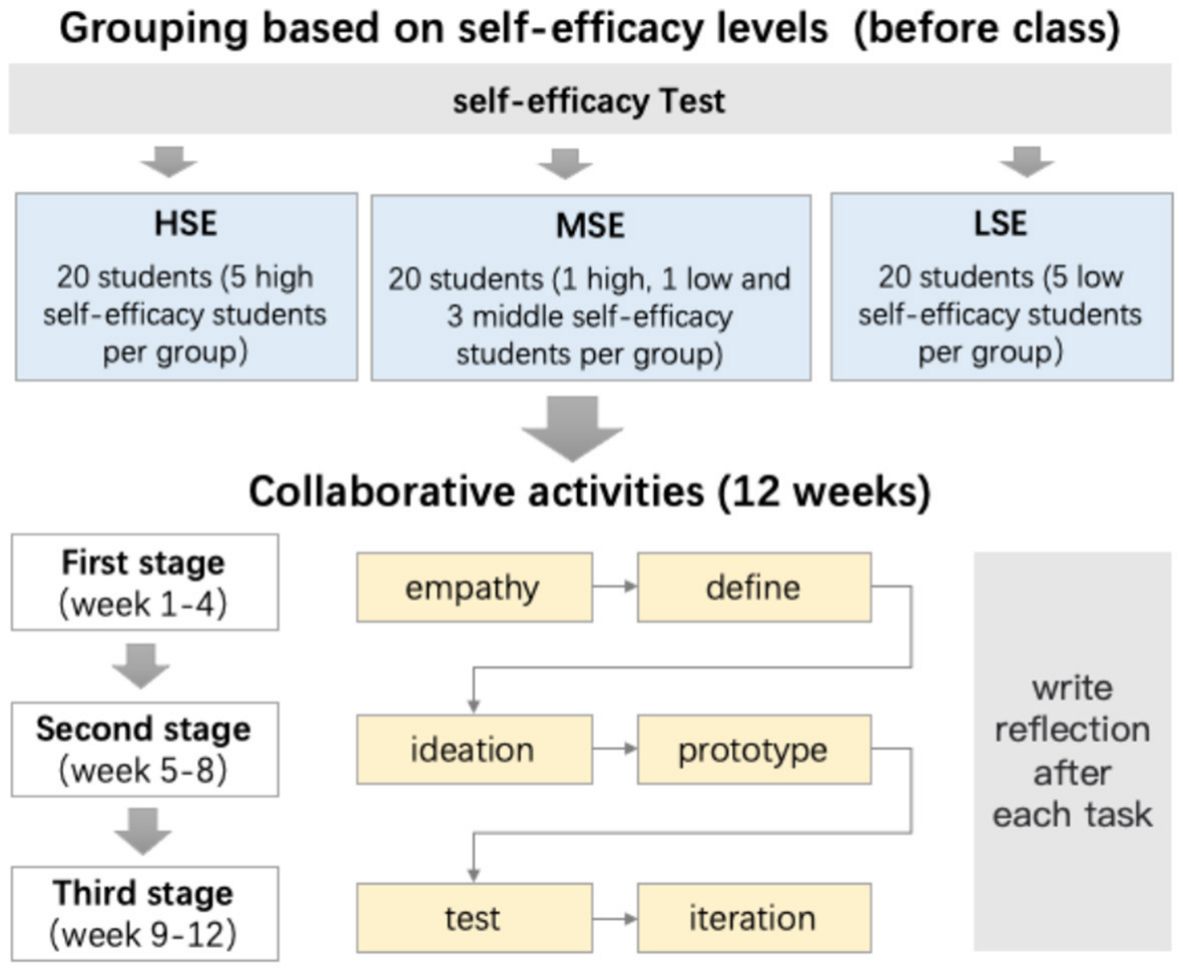
The procedures of the study.

Overall, the study lasted 12 weeks. Figure 1 illustrates the study procedures. Before the class started, participants were required to complete a consent form. They were informed of the purposes and procedure of the study and could quit the study anytime. As a first step of the study, all participants were asked to complete the SE questionnaire adopted by Wang and Lin (2000) at the beginning of the semester. The Motivated Strategies for Learning Questionnaire (MSLQ) by Pintrich et al. (1991) was the source of eight questions on the SE measure, such as “I am confident I can do an excellent job on the assignments and tests in this course.” Wang and Lin (2000) translated the scale into the Chinese version, which has demonstrated reliability with a Cronbach of 0.91 and an acceptable factor structure.

Next, students were grouped based on their SE scores, and they carried out design activities in groups over 12 weeks. The collaborative activity consisted of three stages: inspiration-ideation-realization, which included six sub-tasks: empathy, defining the problem, ideation, prototype, test, and iteration. The six sub-tasks of collaborative activities are elaborated as follows: (1) Empathy. Students develop an understanding of the project context and the actual needs of the user through observation, role play, interview, questionnaire etc. (2) Define. Students extract and identify the problem from the data and information collected in the empathy phase. Then, they deconstruct the task according to the user's needs and convert it into actionable problem-solving steps. (3) Ideation. Students generate diverse, creative ideas and identify and visualize the most feasible solutions. (4) Prototype. With some materials available in daily life, such as Lego, clay, plastic patterns, and hand-made materials, solutions were made into artifacts. (5) Test. To achieve optimal appearance and function, students carry out feasibility tests, prototype functional tests, inter-team tests, extreme user tests, expert tests, etc. (6) Iteration. Students modify the artifacts, and they might even return to the previous stage to look for alternative ideas and solutions.

**Figure 2:**
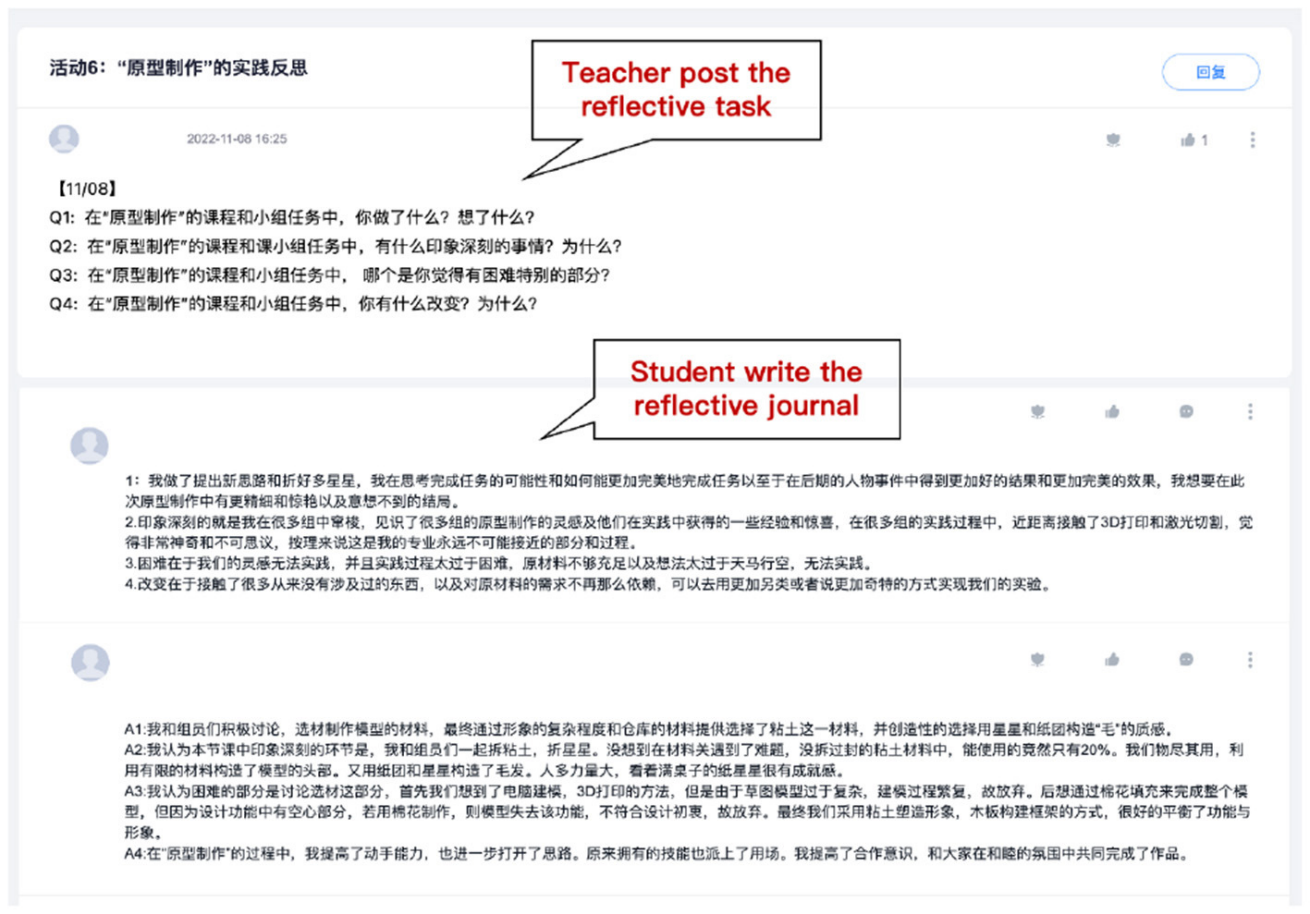
An example of the reflective journal on the learning platform.

Each sub-task lasted for 2 weeks. After each task, students were required to reflect on the process by writing a journal on the learning platform, including the strategies used and the collaborative learning process. Take “Activity 6: reflection of prototype” as an example; students' reflective journals should answer the following questions: Q1: What did you do in the prototype activity? What was in your mind during the activity? Q2: During the prototype activity, what impresses you most? Why? Q3: What is the most difficult part for you in the prototype activity? Q4: What changes have you made in the prototype activity? Why? All the reflections following the same sequence of questions like Activity 6, and a sample reflective journal is shown in Figure 2.

Following Wang and Lin (2007)'s method for grouping participants, we grouped three clusters (i.e., high, low, and mixed) of students based on their scores on the SE questionnaire. The high SE group consisted of students whose SE scores ranked in the top 40%. The low SE group included students' SE scores of the bottom 40%. Finally, there is a mixed SE cluster, where students in the middle 20%, along with four students randomly selected from the high SE cluster and four students randomly chosen from the low SE cluster, were included. Overall, 60 students were assigned to those three clusters. Next, each efficacy cluster was subdivided into four groups, each consisting of five students. Refer to Figure 1 for grouping. The high and low SE groups each had five students with a high or low SE, whereas the mixed SE groups had one student with both a high and low SE and three students with a middle-level SE. After grouping, one-way ANOVA was performed on the SE of learners in the three groups of high (HSE), mixed (MSE) and low (LSE), and the results indicated that there were significant differences in the SE of learners in the three groups (Table 2).

**Table 2:** Descriptive statistics of SE.

**Groups**	***N* (group)**	**Total**	***M***	***SD***	***F* (df)**	***Post-hoc* test**
HSE	4	20	6.688	0.245	87.696^***^	H > M^***^
MSE	4	20	5.888	0.330		M > L^***^
LSE	4	20	5.013	0.266		H > L^***^

HSE, high SE groups; MSE, mixed SE groups; LSE, low SE groups.

^***^p < 0.001.

### 3.4 Coding framework

In our study, students' written reflective journals after each learning task, which include the strategies used in the process of activities and reflection, were used as primary data sources. Overall, this study collected 360 reflective journals of six moves in three stages, totaling 62,570 words.

The reflection journals of 60 students were collected. The journal data was coded to explore the students' SRL among different SE groups in collaborative activities. A coding scheme was developed based on Zimmerman's (2000) three-phase SRL model and Pintrich's (2000) four-stage model of SR, containing three dimensions with a total of 14 subcategories. Table 3 shows the specifics about the dimensions, subcategories, definitions and examples of the coding scheme used in this study. We translated the Chinese reflections into English and presented them as examples in the table.

**Table 3:** Dimensions of coding framework.

**Dimension**	**Subcategories**	**Definition and example**
Forethought	Goal setting (F1)	Sets educational goals or subgoals initially
		Example: “*I decided that the theme of the design goal is to improve the library environment”*.
	Strategic planning (F2)	Plans for sequencing, timing, and completing activities related to goals or subgoals
		Example: “*I planned to go into further investigation in next week”*.
	Task interest/values (F3)	Becomes interested in the task and thinks it has value for him/her
		Example: “*Doing interviews in campus is very interesting”*.
	Self-efficacy (F4)	Describes perceptions of their capabilities to reach a desired outcome
		Example: “*I think I can finish the task”*.
	Outcome expectancies (F5)	Has expectations for problem-solving/task completion
		Example: “*It is hard to transform my ideas into prototypes”*.
Performance	Apply strategies (P1)	Applies strategies, such as interview, observation, role-playing, brainstorming, test, etc.
		Example: “*I used brainstorming to solve problems”*.
	Refine task (P2)	Refines the task while completing the task.
		Example: “*I divided the investigation process into observe, role play, interview etc.”*
	Adjust strategies (P3)	Varies the use of task strategies and adjusts based on outcomes.
		Example: “*When there was not enough clay, I changed materials to make the prototype”*.
	Critical thinking (P4)	Thinks critically about information and tasks
		Example: “*The feasibility of the scheme needs to consider many factors, such as..”*.
	Help-seeking (P5)	Asks peers or teachers for help initially
		Example: “*When it was particularly difficult, I asked my teacher for help.”*
	Heightened interest (P6)	Increases interest in completing the task
		Example: “*I've never been more excited to complete this challenge.”*
Self-reflection	Self-evaluation (S1)	Evaluates the quality or progress of his/her work initially
		Example: “*The way of thinking is obviously different from before”*.
	Causal attribution (S2)	Presents causal attributions about the results of learning efforts
		Example: “*I don't have enough divergent thinking to come up with other innovative solutions.”*
	Self-satisfaction (S3)	Presents perceptions of satisfaction or dissatisfaction (and associated affect) regarding one's performance.
		Example: “*When I saw the final artifacts, I felt very happy and satisfied.”*

### 3.5 Data analysis

The first step of the data analysis is coding. Before doing content analysis, we used Excel to organize the student reflection data. To guarantee the reliability of the coding results, two members of the study team underwent a 2-day coding course. Following the instructions, they coded ten percent of the reflection data at random after sampling. According to Fleiss (1981), the two coders' Cohen's Kappa value was computed to be 0.89, indicating reliability. They then separated the remaining data into two halves and separately coded each of them. The two coders would debate the codes whenever there were disputes or conflicts until they agreed.

To answer the first research question, the first transcripts of students' reflections were coded and the distribution of SRL. For the second and third research questions, the ENA approach was used to investigate the variations in SRL that existed in learning processes among high, low, and mixed SE groups.

We adhered to the analytical methodology and guiding principles suggested by Shaffer et al. (2016). The units of analysis in our study were mixed, low, and high SE groups. To measure the co-occurrence associations of each code, ENA generated an adjacency matrix for each stanza. We searched for semantic relationships between each message and the ones that came before it. According to Sun et al. (2023), each ENA stanza has an adjacency matrix. These matrices are added together to create a cumulative adjacency matrix, which represents epistemic networks. Cognitive elements serve as the nodes in the epistemic network that ENA creates. The frequency of cognitive elements appearing together is represented by the thickness of the linkages between nodes; the stronger the link, the greater the frequency of co-occurrence of cognitive elements (Zhang et al., 2022). Last, we used a two-independent sample *t*-test to analyze and evaluate the distribution of projection points among high, low, and mixed SE groups (e.g., Wu et al., 2023). In addition, we explored the development trajectories of SRL in collaborative learning processes among high, low, and mixed SE groups by analyzing the average networks for the groups in both conditions.

## 4 Results

### 4.1 What are the frequency distribution differences of SRL elements/strategies among different SE groups?

**Figure 3:**
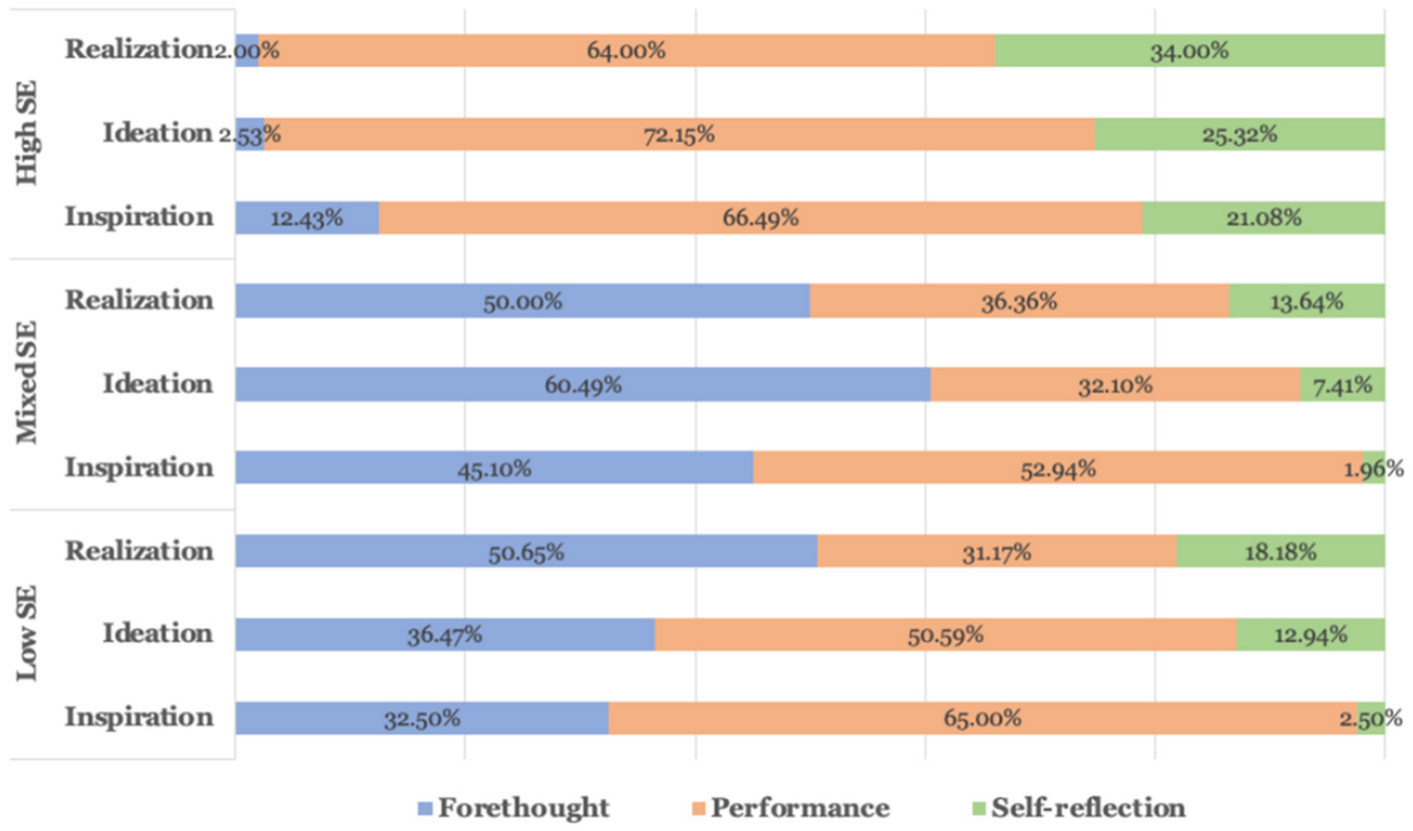
Frequency distribution of SRL strategies in high, mixed, and low SE groups.

To explore the frequency distribution differences of SRL strategies among different SE groups, we conducted a statistical analysis of 1,574 codes from high, mixed, and low SE student groups. As can be seen from Figure 3, the proportions of codes related to the strategies of performance and self-reflection are higher in the high SE groups (66.49, 72.15, 64.00% and 21.08, 25.32, 34.00% in three stages, respectively) than in the mixed (52.94, 32.10, 36.36% and 1.96, 7.41, 13.64% in three stages, respectively) and low SE groups (65.00, 50.59, 31.17% and 2.50, 12.94, 18.18% in three stages, respectively). In terms of the three stages, the proportion of codes related to performance declined in the mixed and low SE groups, and the ratio of codes pertaining to self-reflection in the three groups increased gradually.

To further compare the SRL processes among different SE groups and to consider the temporality, connections and interdependence of self-regulated dimensions, ENA was applied to investigate the SRL patterns of the high, mixed, and low SE groups.

### 4.2 What are the differences in SRL patterns among different SE groups?

**Figure 4:**
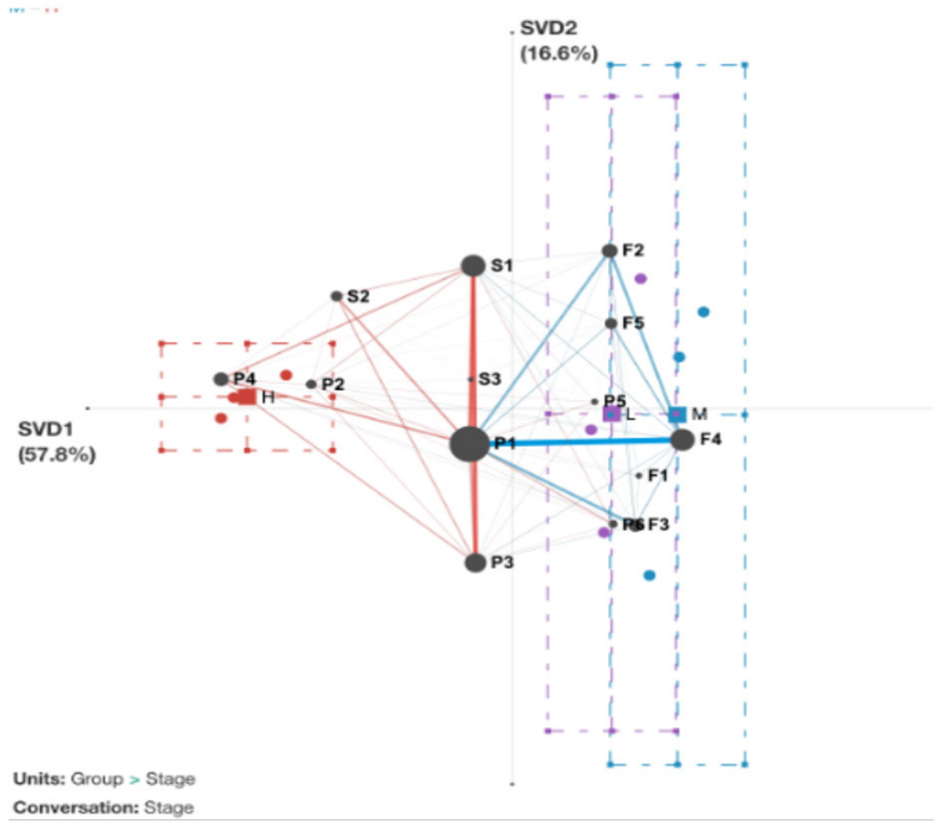
Distribution in SRL patterns among high, mixed, and low SE groups. F1, goal setting; F2, strategic planning; F3, task interest/values; F4, self-efficacy; F5, outcome expectancies; P1, apply strategies; P2, refine task; P3, adjust strategies; P4, critical thinking; P5, help-seeking; P6, heightened interest; S1, self-evaluation; S2, causal attribution; S3, self-satisfaction.

In this study, we analyzed the SRL patterns among three SE student groups. Reflection was taken as the dialogue unit, and the ENA network was used to visualize the SRL mode of learners in the process of collaborative activities in the three groups. Figure 4 shows the differences between learners of different groups. The circles depict centroids, which are the mean positions of the projected points for each network. The rectangles show centroids of the average networks of groups. The red square represents the centroids of the groups with high SE, and the blue square and purple square represent the centroids of the groups with mixed SE and low SE, respectively. There is a notable distance between the centroids of the three groups. The centroid of the high SE group can be found on the left side of the axis, with the centroids of the SE and low SE groups located on the right side. Furthermore, the large and dotted rectangles represent confidence intervals for each group.

**Figure 5:**
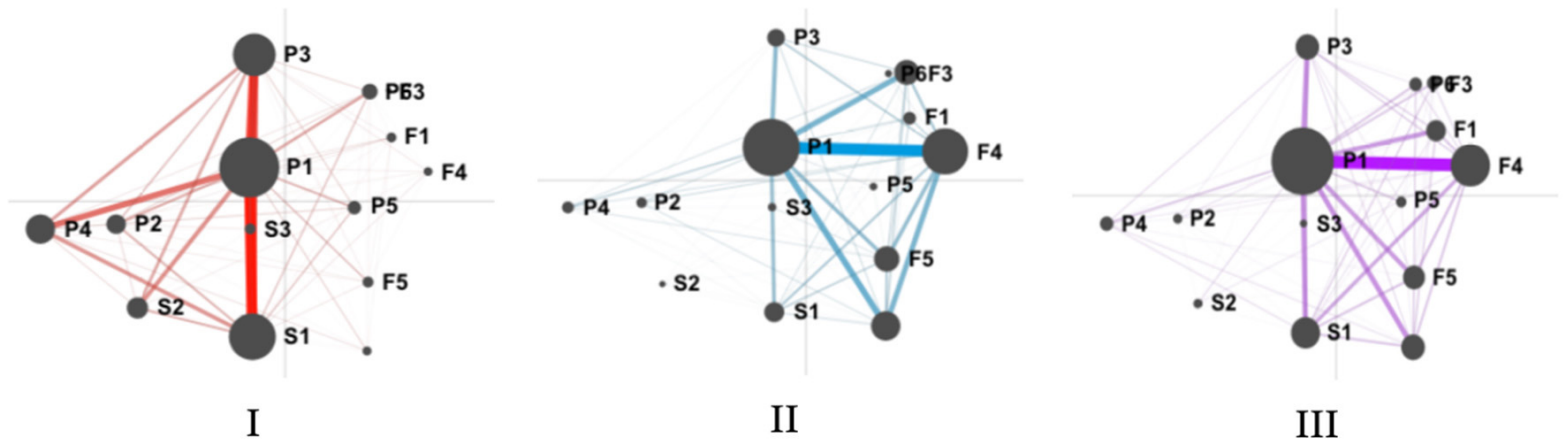
The mean network graphs of high (red), mixed (blue), and low (purple) SE groups. F1, goal setting; F2, strategic planning; F3, task interest/values; F4, self-efficacy; F5, outcome expectancies; P1, apply strategies; P2, refine task; P3, adjust strategies; P4, critical thinking; P5, help-seeking; P6, heightened interest; S1, self-evaluation; S2, causal attribution; S3, self-satisfaction.

Collectively, networks I, II, and III (Figure 5) show that the main difference observed between discussions is along the *X*-axis. SRL behaviors are cognitive elements in this study, which serve as the nodes in the epistemic network that ENA creates. The frequency of cognitive elements appearing together is represented by the thickness of the linkages between nodes. It can be seen from Figure 5 that high SE groups being closer to the left side of the ENA space, which depicted the performance and self-reflection behavior of SRL with apply strategies (P1), self-evaluation (S1), adjust strategies (P3), critical thinking(P4), causal attribution (S2), refine task(P2), seeking help (P5), and self-satisfaction (S3) codes. And the connections between these behaviors are also strong. Mixed and low SE groups are distributed on the right side, which depicts the forethought behavior of SRL with goal setting (F1), planning (F2), SE (F4) and outcome expectation (F5) codes.

In addition, a two-sample *t*-test was used to verify the differences between groups statistically. Table 4 details the differences model between the three levels of high, mixed and low SE. Along the X-axis, the results showed that the high SE group (*M* = −2.73, *SD* = 0.36) was statistically significantly different at the alpha = 0.05 level from the mixed SE group (*M* = 1.70, *SD* = 0.29), *t*(3.78) = −17.00, *p* < 0.001; and low SE group (*M* =1.03, *SD* = 0.27), *t*(3.70) = −14.67, *p* < 0.001. Meanwhile, the difference between mixed and low SE groups was also significant, *t*(3.99) = 3.04, *p* < 0.05. Finally, there was no significant difference along the *Y*-axis.

**Table 4:** Comparison results for the three groups of high, mixed and low SE.

**Comparison group**	**Mean**		***SD***		***T*****-value and** ***p***		***d***	
	**X**	**Y**	**X**	**Y**	**X**	**Y**	**X**	**Y**
HSE	−2.73	0.13	0.36	0.25	*t*(3.78) = −17.00	*t*(2.09) = 0.22	13.88	0.18
MSE	1.70	−0.07	0.28	1.64	*p* = 0.000^***^	*P* = 0.85		
HSE	−2.73	0.13	0.36	0.25	*t*(3.70) = −14.67	*t*(2.11) = 0.22	11.98	0.18
LSE	1.03	−0.06	0.27	1.48	*p* = 0.000^***^	*p* = 0.84		
MSE	1.70	−0.07	0.28	1.64	*t*(3.99) = 3.04	*t*(3.96) = −0.01	2.48	0.01
LSE	1.03	−0.06	0.27	1.48	*p* = 0.04^*^	*p* = 0.99		

HSE, high SE groups; MSE, mixed SE groups; LSE, low SE groups.

^*^p < 0.05, ^***^p < 0.001.

**Figure 6:**
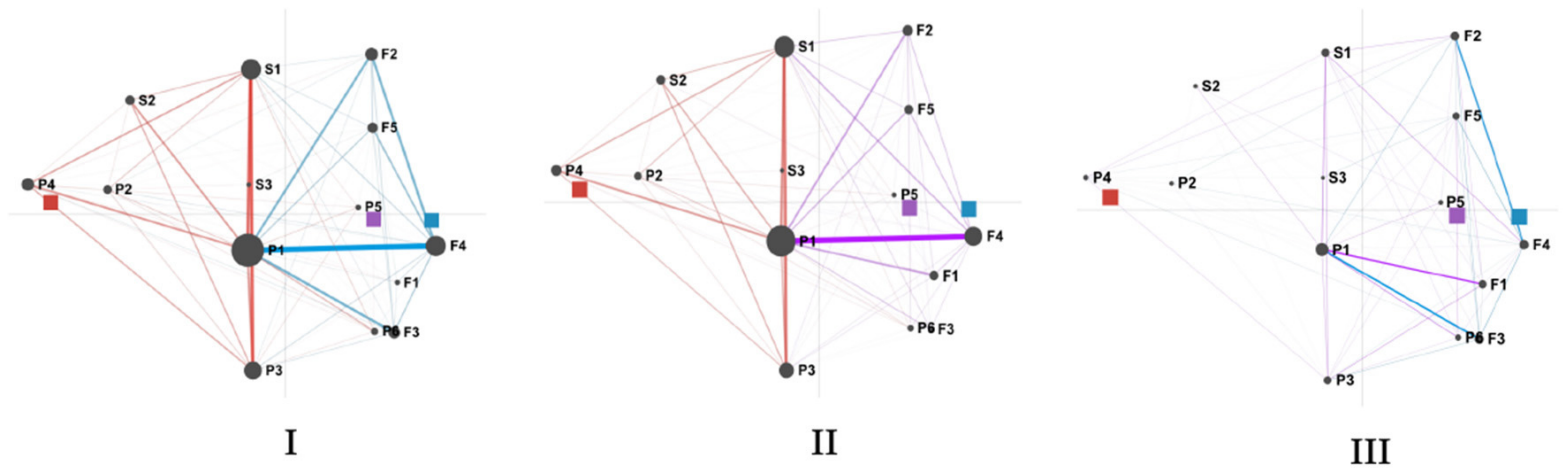
Comparison results of the mean network graphs for high (red), mixed (blue), and low (purple) SE groups. F1, goal setting; F2, strategic planning; F3, task interest/values; F4, SE; F5, outcome expectancies; P1, apply strategies; P2, refine task; P3, adjust strategies; P4, critical thinking; P5, help-seeking; P6, heightened interest; S1, self-evaluation; S2, causal attribution; S3, self-satisfaction.

Figures 4, 5 show the network of the three groups, which provided a summary of the distinctions among discussions. Figure 6 shows subtracted network graphs, which subtract two groups of network nodes and connections from each other to create a different network graph. The network I represents differences between node connections and edge width in high and mixed SE groups. The network II shows differences between node connections and edge width in high and low SE groups. And network III shows differences between node connections and edge width in mixed and low SE groups. Connections with various colors signify that one discussion has stronger linkages between certain codes than the other.

It can be seen from Figure 6 that learners in high SE have more apply strategies (P1)-self-evaluation (S1), apply strategies (P1)-adjust strategies (P3), apply strategies (P1)-critical thinking (P4) connections, while learners in mixed SE have more task interest (F3)-apply strategies (P1), SE (F4)- apply strategies (P1) connections, and learners in low SE have more goal setting (F1)-apply strategies (P1) connections. These differences indicate that compared with learners in the mixed and low SE groups, learners with high SE are more interested in collaborative activities, set challenging goals in the face of problems and believe that the task is solvable. They used more cognitive and metacognitive strategies, reflection and adjustment until they completed the task. However, learners in the mixed-efficacy group and the low-efficacy group were more likely to apply strategies after they planned, found interest, and built confidence for employing strategy and completing the task.

### 4.3 How does the SRL trajectory of different SE groups change in collaborative learning?

**Figure 7:**
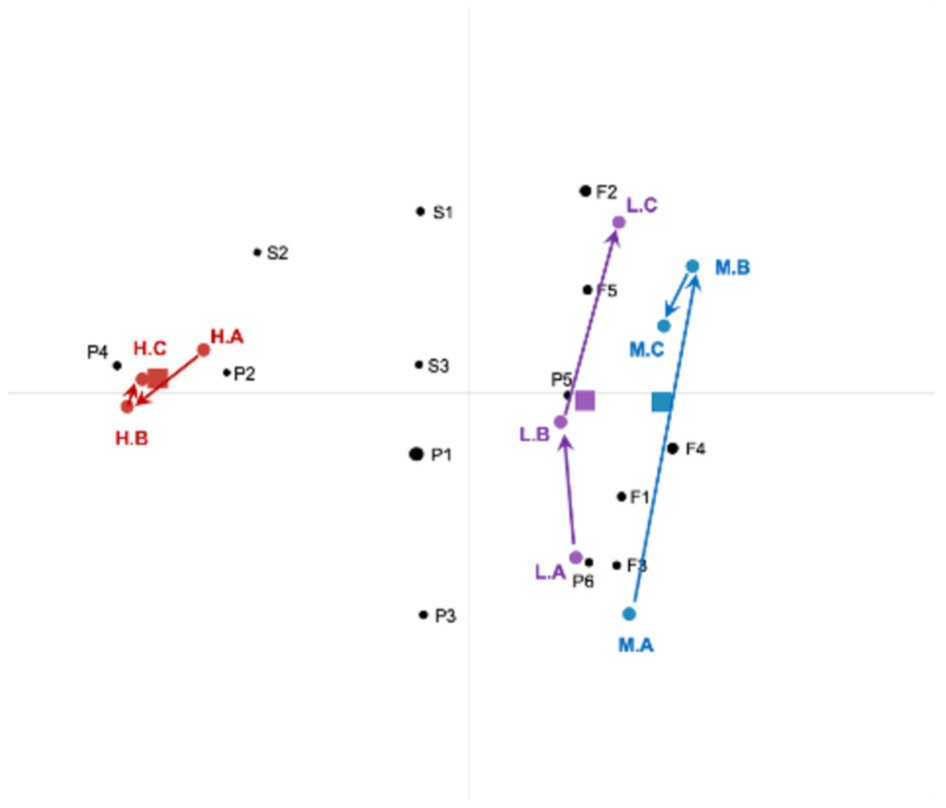
ENA network centroids of the three SE groups in the three learning stages. F1, goal setting; F2, strategic planning; F3, task interest/values; F4, self-efficacy; F5, outcome expectancies; P1, apply strategies; P2, refine task; P3, adjust strategies; P4, critical thinking; P5, help-seeking; P6, heightened interest; S1, self-evaluation; S2, causal attribution; S3, self-satisfaction.

We used ENA to display the centroids of self-regulated learning across three groups in the three learning phases in order to provide more detail on the subtle variations in self-regulated learning patterns among various self-efficacy groups throughout the semester (see Figure 7).

Figure 7 displays the network centroid and the SRL trajectory of the high SE groups using red dots and lines. In contrast, the blue dots and lines represent the network centroid and the SRL trajectory of the mixed SE groups. The purple dots and lines depict the network centroid and the SRL trajectory of the low SE groups.

As shown in Figure 7, the centroids of the high SE groups are mainly observed on the left side of the ENA network space and have mostly stayed the same in the three learning stages. In the first stage, learners apply strategies (P1) and refine tasks (P2). And after a short period of forethought phase, they go into the Performance and Self-reflection phase. In the second stage, learners use critical thinking (P4) to analyze their strategies and activities, while they still conduct critical thinking (P4) and refine task (P2) in the third stage. The trajectory shows that the SRL of high SE learners gradually develops from the forethought to the phase of performance and self-reflection. It may be because learners with high SE have clear learning goals and can constantly self-reflect and adjust strategies while completing tasks.

The centroids of the mixed and low SE groups are mainly observed on the right side of the ENA network space. It can be seen that learners with low SE groups need an effective plan for completing tasks. Learners try to heighten interest (P6) and use different strategies in the first stage, while they constantly ask for help (help-seeking P5) in the second stage. However, the centroids of the third stage move to the forethought phase with strategic planning (F2) and outcome expectancies (F5). Meanwhile, the trajectory of the mixed SE groups had a significant number of linkages related to regulating setting goals, building interest and planning activities during the learning stage.

## 5 Discussion

Using data collected from students' written reflective journals during collaborative design activities, this study investigated the differences in SRL processes among different SE groups by deploying content analysis and ENA. In this section, we discuss our findings and their implications.

### 5.1 Distribution characteristics and patterns of students' SRL process with different SE

In general, the HSE group demonstrated stronger connections between SRL behaviors and were statistically distinct from MSE and LSE (see Figures 3, 4). Specifically, HSE showed more behavioral connections associated with performance and self-regulation, while LSE students exhibited strong links related to forethought. The dense connections to performance and self-regulation made by HSE indicated that students frequently activated metacognitive mechanisms to monitor and regulate the learning process, such as self-evaluation, adjustment strategies, critical thinking and causal attribution. In contrast, learners with low SE spend a lot of time searching for interest or value, determining task objectives, planning and anticipating results, indicating that students with low SE lack SRL strategies and metacognitive mechanisms. According to Schunk and Mullen (2012), individuals with high self-efficacy are more engaged in their work, set more difficult objectives, are more committed to their goals, and think they can overcome obstacles. People with low self-efficacy frequently shy away from difficulties, are less committed to their goals, and see their flaws as personal failings. The higher the SE, the higher the learning performance, and the higher the performance is related to the SE of the next task, which is a positive circular promotion relationship (Wilson and Narayan, 2016). Similar patterns have been found in recent studies on second language learning (e.g., De Backer et al., 2022). From the perspective of social cognitive theory, SE can predict behavior and performance; it can not only positively indicate the use of learning strategies but also affect learners' decision-making, effort, and response to frustration and pressure (Bandura, 1991). Thus, it is unsurprising that students with high SE reported a high level of SRL skills.

Regarding the development of SRL during three phases (forethought, performance, and self-reflection), the ENA model reveals a strong connection between SE and SRL. As indicated in Figure 4, the transitions among apply strategies, self-evaluation and adjust strategies behaviors of high SE students were more frequent than those of the other two groups. According to Bandura (1977) and others such as Boekaerts and Cascallar (2006), efficacy beliefs help learners establish self-regulation mechanisms to guide their performance consistently through shifting conditions. Therefore, it is not surprising that students of HSE displayed more behaviors associated with performance and self-regulation, such as self-evaluation, adjustment strategies, critical thinking, and causal attribution. In addition, when using different strategies, they tend to analyze tasks using critical thinking skills and adapt their strategy for the tasks that follow. Meanwhile, the path of MSE and LSE groups present behaviors such as heightened interest, help-seeking, strategic planning, and outcome expectancies, which means that even at the start of the task (i.e., the performance phase), students of those groups could go back to the forethought phase, spending much time searching for task interest and values, expecting for the outcome and learning from trials and errors, etc. To sum up, students of LSE and MSE are more likely to need more self-reflection strategies, be able to monitor and evaluate their progress and need more self-regulation and meta-cognition skills. Such patterns repeated the studies of Usher and Pajares (2008a), where they found that students with high self-efficacy (SE) utilized more cognitive and metacognitive methods and persisted longer when faced with obstacles compared to those with low self-efficacy. Collectively, our findings are consistent with theoretical perspectives from SE and SRL theorists (Bandura, 1991; Pintrich, 2000; Zimmerman, 2000). Through the analysis of the dynamic changes of SRL of different efficacy groups, we found that the HSE group learners tend to deepen their self-regulation skills as the learning process continues, showing clear development tracks/path of foresight-performance-self-reflection. On the other hand, the self-regulation development trajectory of learners in the LSE group could be more disorderly. Learners are more likely to need more task goals and learning plans and only after continuous use of strategies to explore and try to develop interest in tasks and value recognition.

Collaborative design activities require learners to solve ill-structured problems in real-life settings, which can be challenging. Within the framework of inspiration-ideation-realization, learners need to identify, analyze, and solve the problems. Our ENA analysis found that students of the LSE group manifested a lower level of SRL skills, needed more proper planning, and even the reflection needed to be working more effectively on the strategy adjustment. We also found that students of the MSE group displayed a similar developmental trajectory as the LSE group. Despite it manifesting a phase from planning to reflection, it was a bit vogue, and they could have done better at the task. One possible explanation for this result may be collective efficacy (Bandura, 1991), a term described as a group's shared confidence in its ability to carry out the steps necessary to accomplish a task. Research has demonstrated that an individual's self-efficacy plays a substantial role in their perception of collective efficacy (e.g., Fernández-Ballesteros et al., 2002). In our study, the MSE group consisted of three students of middle-level SE, one student of high SE, and one with low SE. Team members of various levels of SE may end up with a relatively low level of collective efficacy, which negatively impacts the results of teamwork and eventually decreases learners' SE (Bandura et al., 1999). In our study, the student with low SE may end up negatively affected/“dragged” the MSE group. As a result, the MSE group displayed a similar development path as the LSE group.

### 5.2 Implications

#### 5.2.1 Research

This study has contributed to current research in two ways. First, our findings emphasized extant research on self-regulation differences among SE profiles (e.g., Kim et al., 2015). In addition, our work extended current knowledge on the developmental trajectories of SRL among college students in an authentic, collaborative learning sessions. Understanding the patterns and paths of the regulation process provides teachers and stakeholders alike a picture regards how well students have prepared, executed, and reflected during a task, thus offering follow-up intervention to prevent future failure.

Secondly, to date, this study is one of the early attempts that apply the ENA approach to understand the complex self-regulation process during collaborative design activities. Despite current interest in understanding the complex interaction of the self-regulation process and SE across different disciplinaries and educational levels (Chen et al., 2022), the majority of extant studies used (traditional) coding-and-counting approaches, thus the temporal development of the regulation process was ignored to a large extent. Merits of ENA, such as enabling the exploration of the temporal co-occurrences of codes, provide a more appropriate way to characterize socio-cognitive activities of learning in collaborative learning settings (Shaffer et al., 2016; Csanadi et al., 2018). Thanks to ENA, we were able to capture the group's differences and the subtle, dynamic changes when using self-regulation strategies. Based on the findings, it is possible to provide individualized remedies/interventions for students, for instance, providing clear feedback technology support and scaffolding to low-efficacy students. Moreover, how students with different SE levels can be guided to design thinking and regulate their learning and to do during this thinking? For example, students with low SE may need special support; what is the best supporting strategy for them? Those can be interesting topics for future study.

#### 5.2.2 Practice

The results of this study have implications for how teachers can plan and execute lessons in the classroom to help students become more adept at self-regulation. First and foremost, as students' SE is most strongly correlated with their use of SRL strategies, it is imperative to support their SE. Since SE is concerned with students' confidence in their ability to complete certain activities, teachers should provide each student with constructive and detailed feedback for improvements. Teachers can also help students grow confidence by offering additional support and guidance. Moreover, extant research has proved that trust between partners, peer support, and group cohesion are crucial to boosting SE (Alavi and McCormick, 2008; Sun and Lin, 2022). Specifically, trust is associated with various factors such as individual member performance, team performance, and creative problem-solving (Hacker et al., 2019). Meanwhile, current studies also found several inhibitors (e.g., language and cultural differences, communication, and technology usage) in building trust within the group (Cheng et al., 2016b; Hacker et al., 2019). However, such negative impacts could be mitigated through a collaboration process and clear task design (Cheng et al., 2016a,b). Therefore, teachers can arrange collaborative activities in a way that builds trust within the group, maximize the remodeling of high-efficacy learners while minimizing the negative effect of low-efficacy students, and promotes healthy communication and interaction among group members, which, in turn, helps to build students' self- and collective efficacy. Last, our study has found that for LSE and MSE students were more likely to linger around the phrase “forethought” and it was difficult for them to use self-regulation strategies properly. Thus, providing and supporting students with the right tools and scaffolding are equally important. For instance, teachers may use software tools to facilitate information processing by giving cognitive scaffolds for learning strategy use (Malmberg et al., 2010; Perry et al., 2010), applying visualization tools that monitor learning progress, and using mirroring tools to enhance awareness of the collaborative learning process (Fransen et al., 2011).

According to our results, different regulation process patterns were identified for various levels of SE. One distinguishing feature is that, among the learners of low and mixed levels of SE, they spent around 30–60% of their effort lingering around the forethought phase. So, how to support them to take the next step? It might be difficult for low SE students to go ahead and start generating ideas or design solutions since the emphasis of generating ideas/or design solutions is on divergent thinking and seeing beyond the obvious. Students use creativity to investigate, search for, and produce ideas and to experiment and manipulate unconventional, risky or innovative ideas (Vincent-Lancrin et al., 2019). For students of a higher level of efficacy, focus on their performance stage; for learners that need more confidence in their capabilities, try to provide in time support even at the preparation (forethought) phase. Therefore, formal methods that are supportive of ideation or generation of ideas are needed. For example, before each task session, teachers can guide students to do presentations that repost the process they made and plan in a way to avoid lingering around activities such as searching for task value and reflect their process.

### 5.3 Limitations and future directions

Several limitations were present in the current study that could be rectified in future research. First, this study investigated the characteristics and developmental patterns of SRL in collaborative learning from an individual level. Recently, the concepts of co-regulation (i.e., learners' SR on their own with peer support via social interactions) and socially shared regulation (i.e., the deliberate, strategic and transactive planning; task performance; and reflection of a group) have been recognized as other forms of social regulation in collaborative learning settings (Järvelä et al., 2018; Nguyen et al., 2023), based on the assumption that collaborative learning regulation exists on a spectrum between individual and societal aspects (Malmberg et al., 2015). Thus, future studies on collaborative learning research are suggested to aim at both individual and group/social levels to figure out the complex mechanism and contribution of within and between levels on task performance and SRL skills. For instance, explore the roles of socially shared regulation, emotional interaction on collaborative learning performance and temporal variety in using SRL strategies among different student groups.

Second, this study needs more datasets. We used students' written self-reflection during the collaborative design activities. Although this approach can provide rich information regarding the developmental path of learners, it can be limited when systematically understanding the complicated process of regulation of cognition, motivation, and emotion in a given task. More valid data, such as data accessed from eye tracking, observation, video recordings, and critical interviews, can be used in future studies to capture learners' unfolding regulation process across various authentic tasks and situations and address the limitations of conventional single-channel data in order to assist in making more accurate and trustworthy inferences on the learning processes (Harley et al., 2015). In addition, as also posited by Järvenoja et al. (2019), further evidence is needed to ascertain the insights provided by various modalities (e.g., electrodermal activity, heart rate) on learning and collaboration processes.

## 6 Conclusion

The current study investigated the patterns and developmental trajectories of university students of various efficacy levels (i.e., high, mixed, and low) in an authentic, collaborative learning setting. Epistemic network analysis was used to capture and compare the dynamic changes and paths of the regulation process across groups. There are two important takeaways based on the results: First, high-efficacy students are more likely to recognize task interest and value and use advanced regulation strategies such as focusing on performance and regulation; they tend to display a clear “anticipation-behavior-reflection” SRL path; Second, students of lower SE are more likely to linger around the initial phase “forethought” and present a bit messy path of regulation process. Collectively, our study has contributed to the current understanding of the role of SE on the complex developmental trajectories of regulation in college-level education. Accordingly, strategies and suggestions for improving learners' SRL in collaborative design activities were proposed.

## Data availability statement

The raw data supporting the conclusions of this article will be made available by the authors, without undue reservation.

## Ethics statement

The studies involving humans were approved by the Local Ethics Committee of Capital Normal University. The studies were conducted in accordance with the local legislation and institutional requirements. The participants provided their written informed consent to participate in this study.

## Author contributions

PC: Writing – original draft, Visualization, Methodology, Funding acquisition, Conceptualization. DY: Writing – review & editing, Visualization, Methodology, Conceptualization. JL: Writing – review & editing. AHSM: Writing – review & editing. XT: Writing – review & editing.

## Appendix 1

The SE questionnaire adopted by Wang and Lin (2000)

(1) I believe I will receive an excellent grade in this class.
(2) I'm certain I can understand the most difficult material presented in the readings for this course.
(3) I'm confident I can understand the basic concepts taught in this course.
(4) I'm confident I can understand the most complex material presented by the instructor in this course.
(5) I'm confident I can do an excellent job on the assignments and tests in this course.
(6) I expect to do well in this class.
(7) I'm certain I can master the skills being taught in this class.
(8) Considering the difficulty of this course, the teacher, and my skills, I think I will do well in this class.

## References

[B1] Aguado D. Rico R. Sánchez-Manzanares M. Salas E. (2014). Teamwork competency test (TWCT): a step forward on measuring teamwork competencies. Group Dyn. Theory Res. Pract. 18, 101–121. 10.1037/a0036098

[B2] Alavi S. B. McCormick J. (2008). The roles of perceived task interdependence and group members' interdependence in the development of collective efficacy in university student group contexts. Br. J. Educ. Psychol. 78, 375–393. 10.1348/000709907X240471 17845739

[B3] An S. Zhang S. (2024). Effects of ability grouping on students' collaborative problem solving patterns: evidence from lag sequence analysis and epistemic network analysis. Think. Skills Creat. 51:101453. 10.1016/j.tsc.2023.101453

[B4] Bai B. Wang J. Nie Y. (2021). Self-efficacy, task values and growth mindset: what has the most predictive power for primary school students' self-regulated learning in English writing and writing competence in an Asian Confucian cultural context? Camb. J. Educ. 51, 65–84. 10.1080/0305764X.2020.1778639

[B5] Bandura A. (1977). Social Learning Theory. Oxford: Prentice-Hall.

[B6] Bandura A. (1991). Social cognitive theory of self-regulation. Organ. Behav. Hum. Decis. Process. 50, 248–287. 10.1016/0749-5978(91)90022-L

[B7] Bandura A. Freeman W. H. Lightsey R. (1999). Self-efficacy: the exercise of control. J. Cogn. Psychother. 13, 158–166. 10.1891/0889-8391.13.2.15811261958

[B8] Boekaerts M. Cascallar E. (2006). How far have we moved toward the integration of theory and practice in self-regulation? Educ. Psychol. Rev. 18, 199–210. 10.1007/s10648-006-9013-4

[B9] Boekaerts M. Niemivirta M. (2000). “Self-regulated learning: Finding a balance between learning goals and ego-protective goals,” in Handbook of Self-Regulation, eds. M. Boekaerts, P. R. Pintrich, and M. Zeidner (San Diego, CA: Academic Press), 417–450.

[B10] Brown T. (2008). Design thinking. Harv. Bus. Rev. 86, 84–92. 18605031

[B11] Chen J. Zhang L. J. Chen X. (2022). L2 learners' self-regulated learning strategies and self-efficacy for writing achievement: a latent profile analysis. Lan. Teach. Res. 10.1177/13621688221134967

[B12] Chen Y. Li C. Cao L. Liu S. (2024). The effects of self-efficacy, academic stress, and learning behaviors on self-regulated learning in blended learning among middle school students. Educ. Inf. Technol. 10.1007/s10639-024-12821-w

[B13] Cheng X. Fu S. Druckenmiller D. (2016a). Trust development in globally distributed collaboration: a case of U.S. and Chinese Mixed Teams. J. Manag. Inf. Syst. 33, 978–1007. 10.1080/07421222.2016.1267521

[B14] Cheng X. Fu S. Sun J. Han Y. Shen J. Zarifis A. (2016b). Investigating individual trust in semi-virtual collaboration of multicultural and unicultural teams. Comput. Human Behav. 62, 267–276. 10.1016/j.chb.2016.03.093

[B15] Cleary T. J. Velardi B. Schnaidman B. (2017). Effects of the self-regulation empowerment program (SREP) on middle school students' strategic skills, self-efficacy, and mathematics achievement. J. Sch. Psychol. 64, 28–42. 10.1016/j.jsp.2017.04.004 28735606

[B16] Cleary T. J. Zimmerman B. J. Keating T. (2006). Training physical education students to self-regulate during basketball free throw practice. Res. Q. Exerc. Sport 77, 251–262. 10.1080/02701367.2006.10599358 16898280

[B17] Csanadi A. Eagan B. Kollar I. Shaffer D. W. Fischer F. (2018). When coding-and-counting is not enough: using epistemic network analysis (ENA) to analyze verbal data in CSCL research. Int. J. Comp. Support. Collab. Learn. 13, 419–438. 10.1007/s11412-018-9292-z

[B18] De Backer L. Van Keer H. De Smedt F. Merchie E. Valcke M. (2022). Identifying regulation profiles during computer-supported collaborative learning and examining their relation with students' performance, motivation, and self-efficacy for learning. Comp. Educ. 179:104421. 10.1016/j.compedu.2021.104421

[B19] DiBenedetto M. K. Bembenutty H. (2013). Within the pipeline: self-regulated learning, self-efficacy, and socialization among college students in science courses. Learn. Individ. Differ. 23, 218–224. 10.1016/j.lindif.2012.09.015

[B20] Dignath C. Buettner G. Langfeldt H.-P. (2008). How can primary school students learn self-regulated learning strategies most effectively? Educ. Res. Rev. 3, 101–129. 10.1016/j.edurev.2008.02.003

[B21] Diseth Å. (2011). Self-efficacy, goal orientations and learning strategies as mediators between preceding and subsequent academic achievement. Learn. Individ. Differ. 21, 191–195. 10.1016/j.lindif.2011.01.003

[B22] Dunne D. Martin R. (2006). Design thinking and how it will change management education: an interview and discussion. Acad. Manag. Learn. Educ. 5, 512–523. 10.5465/amle.2006.23473212

[B23] Efklides A. (2011). Interactions of metacognition with motivation and affect in self-regulated learning: the MASRL model. Educ. Psychol. 46, 6–25. 10.1080/00461520.2011.538645

[B24] Elmoazen R. Saqr M. Tedre M. Hirsto L. (2022). A systematic literature review of empirical research on epistemic network analysis in education. IEEE Access 10, 17330–17348. 10.1109/ACCESS.2022.314981236039351

[B25] EU Council (2002). Council Resolution of 27 June 2002 on Lifelong Learning. Available online at: https://op.europa.eu/en/publication-detail/-/publication/0bf0f197-5b35-4a97-9612-19674583cb5b/language-en (accessed March 30, 2024).

[B26] Fernández-Ballesteros R. Díez-Nicolás J. Caprara G. V. Barbaranelli C. Bandura A. (2002). Determinants and structural relation of personal efficacy to collective efficacy. Appl. Psychol. 51, 107–125. 10.1111/1464-0597.00081

[B27] Fleiss J. L. (1981). Statistical Methods for Rates and Proportions (2nd ed.). London: John Wiley.

[B28] Fraile J. Gil-Izquierdo M. Medina-Moral E. (2023). The impact of rubrics and scripts on self-regulation, self-efficacy and performance in collaborative problem-solving tasks. Assess. Eval. High. Educ. 48, 1223–1239. 10.1080/02602938.2023.2236335

[B29] Fransen J. Kirschner P. A. Erkens G. (2011). Mediating team effectiveness in the context of collaborative learning: the importance of team and task awareness. Comput. Human Behav. 27, 1103–1113. 10.1016/j.chb.2010.05.017

[B30] Garrison D. Anderson T. Archer W. (2001). Critical thinking, cognitive presence, and computer conferencing in distance education. Am. J. Dist. Educ. 15, 7–23. 10.1080/0892364010952707129118067

[B31] Gašević D. Joksimović S. Eagan B. R. Shaffer D. W. (2019). SENS: network analytics to combine social and cognitive perspectives of collaborative learning. Comput. Human Behav. 92, 562–577. 10.1016/j.chb.2018.07.003

[B32] Gunawardena C. Lowe C. Anderson T. (1997). Analysis of a global online debate and the development of an interaction analysis model for examining social construction of knowledge in computer conferencing. J. Educ. Comp. Res. 4, 397–431. 10.2190/7MQV-X9UJ-C7Q3-NRAG22612255

[B33] Hacker J. V. Johnson M. Saunders C. Thayer A. L. (2019). Trust in virtual teams: a multidisciplinary review and integration. Aust. J. Inf. Syst. 23:1757. 10.3127/ajis.v23i0.1757

[B34] Hadwin A. Bakhtiar A. Miller M. (2018). Challenges in online collaboration: effects of scripting shared task perceptions. Int. J. Comp. Support. Collab. Learn. 13, 301–329. 10.1007/s11412-018-9279-9

[B35] Harley J. M. Bouchet F. Hussain M. S. Azevedo R. Calvo R. (2015). A multi-componential analysis of emotions during complex learning with an intelligent multi-agent system. Comput. Human Behav. 48, 615–625. 10.1016/j.chb.2015.02.013

[B36] Harris K. R. Graham S. (2009). Self-regulated strategy development in writing: Premises, evolution, and the future. Br. J. Educ. Psychol. 2, 113–135. 10.1348/978185409X422542

[B37] Hassi L. Laakso M. (2011). Design Thinking in the Management Discourse: Defining the Elements of the Concept. Delft University of Technology, Delft, 1–14.

[B38] Järvelä S. Hadwin A. Malmberg J. Miller M. (2018). “Contemporary perspectives of regulated learning in collaboration,” in International Handbook of the Learning Sciences, eds. F. Fischer, C. E. Hmelo-Silver, P. Reimann, and S. R. Goldman (New York, NY: Routledge), 127–136.

[B39] Järvelä S. Järvenoja H. Malmberg J. (2019). Capturing the dynamic and cyclical nature of regulation: methodological Progress in understanding socially shared regulation in learning. Int. J. Comp. Support. Collab. Learn. 14, 425–441. 10.1007/s11412-019-09313-2

[B40] Järvenoja H. Näykki P. Törmänen T. (2019). Emotional regulation in collaborative learning: when do higher education students activate group level regulation in the face of challenges? Stud. High. Educ. 44, 1747–1757. 10.1080/03075079.2019.1665318

[B41] Kim D.-H. Wang C. Ahn H. S. Bong M. (2015). English language learners' self-efficacy profiles and relationship with self-regulated learning strategies. Learn. Individ. Differ. 38, 136–142. 10.1016/j.lindif.2015.01.016

[B42] Laal M. Laal M. (2012). Collaborative learning: what is it? Proc. Soc. Behav. Sci. 31, 491–495. 10.1016/j.sbspro.2011.12.092

[B43] Luka I. (2014). Design thinking in pedagogy. J. Educ. Cult. Soc. 5, 63–74. 10.15503/jecs20142.63.74

[B44] Magogwe J. M. Oliver R. (2007). The relationship between language learning strategies, proficiency, age and self-efficacy beliefs: a study of language learners in Botswana. System 35, 338–352. 10.1016/j.system.2007.01.003

[B45] Malmberg J. Järvelä S. Järvenoja H. Panadero E. (2015). Promoting socially shared regulation of learning in CSCL: progress of socially shared regulation among high- and low-performing groups. Comput. Human Behav. 52, 562–572. 10.1016/j.chb.2015.03.082

[B46] Malmberg J. Järvenoja H. Järvelä S. (2010). Tracing elementary school students' study tactic use in gStudy by examining a strategic and self-regulated learning. Comput. Human Behav. 26, 1034–1042. 10.1016/j.chb.2010.03.004

[B47] Melles G. Misic V. (2011). Introducing design thinking to undergraduate students at Swinburn university. Jpn. Soc. Sci. Des. 20, 2–7. 10.11247/jssds.20.1_2

[B48] Moghadari-Koosha M. Moghadasi-Amiri M. Cheraghi F. Mozafari H. Imani B. Zandieh M. (2020). Self-efficacy, self-regulated learning, and motivation as factors influencing academic achievement among paramedical students: a correlation study. J. Allied Health 49, e145–e152. 32877483

[B49] Nguyen A. Järvelä S. Rosé C. Järvenoja H. Malmberg J. (2023). Examining socially shared regulation and shared physiological arousal events with multimodal learning analytics. Br. J. Educ. Technol. 54, 293–312. 10.1111/bjet.13280

[B50] Nokes-Malach T. J. Richey J. E. Gadgil S. (2015). When is it better to learn together? Insights from research on collaborative learning. Educ. Psychol. Rev. 27, 645–656. 10.1007/s10648-015-9312-8

[B51] Noroozi O. Järvelä S. Kirschner P. A. (2019). Multidisciplinary innovations and technologies for facilitation of self-regulated learning. Comput. Human Behav. 100, 295–297. 10.1016/j.chb.2019.07.020

[B52] Panadero E. (2017). A review of self-regulated learning: six models and four directions for research. Front. Psychol. 8:422. 10.3389/fpsyg.2017.00422 28503157 PMC5408091

[B53] Paraskeva F. (2007). Self-regulated learning strategies and computer self-efficacy in IT courses. WIT Transact. Inf. Commun. Technol. 38:231. 10.2495/DATA07023137361755

[B54] Perry N. Carolyn T. Hutchinson L. (2010). gStudy traces of children's self-regulated learning in the Lifecycles Learning Kit. Psychol. Test Assess. Model. 52:432.

[B55] Pintrich P. R. (2000). “The role of goal orientation in self-regulated learning,” in Handbook of Self-Regulation, eds. M. Boekaerts, P. R. Pintrich, and M. Zeidner (San Diego, CA: Academic Press), 451–502.

[B56] Pintrich P. R. Smith D. Garcia T. McKeachie W. (1991). The Motivated Strategies for Learning Questionnaire (MSLQ). Ann Arbor: MI: NCRIPTAL, The University of Michigan. Available online at: https://eric.ed.gov/?id=ED338122 (accessed September 11, 2022).

[B57] Robson D. A. Allen M. S. Howard S. J. (2020). Self-regulation in childhood as a predictor of future outcomes: a meta-analytic review. Psychol. Bull. 146, 324–354. 10.1037/bul0000227 31904248

[B58] Schunk D. H. Mullen C. A. (2012). “Self-efficacy as an engaged learner,” in Handbook of Research on Student Engagement, eds. S. Christenson, A. Reschly, and C. Wylie (Boston, MA: Springer), 219–235.

[B59] Schunk D. H. Zimmerman B. J. (2013). “Self-regulation and learning,” in Handbook of Psychology: Educational Psychology, Vol. 7, 2nd Edn, eds. W. M. Reynolds, G. E. Miller, and L. B. Weiner (Hoboken, NJ: John Wiley and Sons, Inc.), 45–68.

[B60] Shaffer D. W. Collier W. Ruis A. R. (2016). A tutorial on epistemic network analysis: analyzing the structure of connections in cognitive, social, and interaction data. J. Learn. Anal. 3, 9–45. 10.18608/jla.2016.33.3

[B61] Shen B. Bai B. (2022). Chinese university students' self-regulated writing strategy use and EFL writing performance: influences of self-efficacy, gender, and major. Appl. Linguist. Rev. 15, 161–188. 10.1515/applirev-2020-0103

[B62] Simon H. (1969). The Sciences of the Artifcial. Cambridge: The MIT Press.

[B63] Sun J. C.-Y. Lin H.-S. (2022). Effects of integrating an interactive response system into flipped classroom instruction on students' anti-phishing self-efficacy, collective efficacy, and sequential behavioral patterns. Comp. Educ. 180:104430. 10.1016/j.compedu.2022.104430

[B64] Sun Z. Xu R. Deng L. Jin F. Song Z. Lin C.-H. (2023). Beyond coding and counting: exploring teachers' practical knowledge online through epistemic network analysis. Comp. Educ. 192:104647. 10.1016/j.compedu.2022.104647

[B65] Teng M. F. Wang C. Zhang L. J. (2022). Assessing self-regulatory writing strategies and their predictive effects on young EFL learners' writing performance. Assess. Writing 51:100573. 10.1016/j.asw.2021.100573

[B66] Usher E. L. Pajares F. (2008a). Self-efficacy for self-regulated learning: a validation study. Educ. Psychol. Meas. 68, 443–463. 10.1177/0013164407308475

[B67] Usher E. L. Pajares F. (2008b). Sources of self-efficacy in school: critical review of the literature and future directions. Rev. Educ. Res. 78, 751–796. 10.3102/003465430832145638293548

[B68] Vandevelde S. Van Keer H. Schellings G. Van Hout-Wolters B. (2015). Using think-aloud protocol analysis to gain in-depth insights into upper primary school children's self-regulated learning. Learn. Individ. Differ. 43, 11–30. 10.1016/j.lindif.2015.08.027

[B69] Vincent-Lancrin S. Gonzalez-Sancho C. Bouckaert M. de Luca F. Fernández-Barrerra M. Jacotin G.. (2019). “Creativity and critical thinking in everyday teaching and learning,” in Fostering Students' Creativity and Critical Thinking (Paris: OECD Publishing), 127–164.

[B70] Wang S.-L. Lin S. (2000). “The cross-cultural validation of motivated strategies for learning questionnaire,” in Paper presented at the 2000 Annual Conference of American Psychological Association (Washington, DC).

[B71] Wang S.-L. Lin S. (2007). The effects of group composition of self-efficacy and collective efficacy on computer-supported collaborative learning. Comput. Human Behav. 23, 2256–2268. 10.1016/j.chb.2006.03.005

[B72] Wang T. Li S. Tan C. Zhang J. Lajoie S. P. (2023). Examining the relationship between cognitive load patterns and self-regulated learning within a technology-rich learning environment. Comp. Educ. 26:104924. 10.1016/j.compedu.2023.104924

[B73] Wang Y. (2023). Enhancing English reading skills and self-regulated learning through online collaborative flipped classroom: a comparative study. Front. Psychol. 14:1255389. 10.3389/fpsyg.2023.1255389 37908818 PMC10613993

[B74] Wilson K. Narayan A. (2016). Relationships among individual task self-efficacy, self-regulated learning strategy use and academic performance in a computer-supported collaborative learning environment. Educ. Psychol. 36, 236–253. 10.1080/01443410.2014.926312

[B75] Winne P. H. Hadwin A. F. (2008). “The weave of motivation and self-regulated learning,” in Motivation and Self-Regulated Learning: Theory, Research, and Applications, eds. D.H. Schunk and B. J. Zimmerman (Mahwah, NJ: Lawrence Erlbaum Associates Publishers), 297–314.

[B76] Winne P. H. Jamieson-Noel D. Muis K. (2002). Methodological issues and advances in researching tactics, strategies, and self-regulated learning. Adv. Motiv. Achiev. 12, 121–155.

[B77] Wu L. Yu S. Liu Q. Ye J. Zheng X. Wang J. (2023). Is cross-discipline better than same-discipline for cognitive engagement in computer supported collaborative learning? An empirical study using epistemic network analysis. J. Comp. High. Educ. 10.1007/s12528-023-09389-8

[B78] Yun S. Hiver P. Al-Hoorie A. (2018). Academic buoyancy: exploring learners' everyday resilience in the language classroom. Stud. Sec. Lang. Acquisit. 40, 805–830. 10.1017/S0272263118000037

[B79] Zhang S. Chen J. Wen Y. Chen H. Gao Q. Wang Q. (2021). Capturing regulatory patterns in online collaborative learning: a network analytic approach. Int. J. Comp. Support. Collab. Learn. 16, 37–66. 10.1007/s11412-021-09339-5

[B80] Zhang S. Gao Q. Sun M. Cai Z. Li H. Tang Y.. (2022). Understanding student teachers' collaborative problem solving: Insights from an epistemic network analysis (ENA). Comp. Educ. 183:104485. 10.1016/j.compedu.2022.104485

[B81] Zhao S. Cao C. (2023). Exploring relationship among self-regulated learning, self-efficacy and engagement in blended collaborative context. SAGE Open 13:215824402311572. 10.1177/21582440231157240

[B82] Zimmerman B. Schunk D. (2008). “Motivation: An essential dimension of self-regulated learning,” in Motivation and Self-Regulated Learning: Theory, Research, and Applications, eds. D. Schunk and B. Zimmerman (New York, NY: Routledge), 1–30.

[B83] Zimmerman B. J. (2000). “Attaining self-regulation: a social cognitive perspective,” in Handbook of Self-Regulation, eds. M. Boekaerts, P. R. Pintrich, and M. Zeidner (San Diego, CA: Academic Press), 13–39.

[B84] Zimmerman B. J. (2013). From cognitive modeling to self-regulation: a social cognitive career path. Educ. Psychol. 48, 135–147. 10.1080/00461520.2013.794676

